# Portable TDLAS Sensor for Online Monitoring of CO_2_ and H_2_O Using a Miniaturized Multi-Pass Cell

**DOI:** 10.3390/s23042072

**Published:** 2023-02-12

**Authors:** Mingsi Gu, Jiajin Chen, Yiping Zhang, Tu Tan, Guishi Wang, Kun Liu, Xiaoming Gao, Jiaoxu Mei

**Affiliations:** 1 Anhui Institute of Optics and Fine Mechanics, Hefei Institutes of Physical Science, Chinese Academy of Sciences, Hefei 230031, China; 2 Science Island Branch of Graduate School, University of Science and Technology of China, Hefei 230026, China; 3 Anhui Advanced Spectroscopy Optical-Electric S&T Co., Ltd., Hefei 230026, China

**Keywords:** tunable diode laser absorption spectroscopy (TDLAS), online monitoring, CO_2_, H_2_O, multi-pass cell (MPC), distributed feedback (DFB) laser, portable system

## Abstract

We designed a tunable diode laser absorption spectroscopy (TDLAS) sensor for the online monitoring of CO_2_ and H_2_O concentrations. It comprised a small self-design multi-pass cell, home-made laser drive circuits, and a data acquisition circuit. The optical and electrical parts and the gas circuit were integrated into a portable carrying case (height = 134 mm, length = 388 mm, and width = 290 mm). A TDLAS drive module (size: 90 mm × 45 mm) was designed to realize the function of laser current and temperature control with a temperature control accuracy of ±1.4 mK and a current control accuracy of ±0.5 μA, and signal acquisition and demodulation. The weight and power consumption of the TDLAS system were only 5 kg and 10 W, respectively. Distributed feedback lasers (2004 nm and 1392 nm) were employed to target CO_2_ and H_2_O absorption lines, respectively. According to Allan analysis, the detection limits of CO_2_ and H_2_O were 0.13 ppm and 3.7 ppm at an average time of 18 s and 35 s, respectively. The system response time was approximately 10 s. Sensor performance was verified by measuring atmospheric CO_2_ and H_2_O concentrations for 240 h. Experimental results were compared with those obtained using a commercial instrument LI-7500, which uses non-dispersive infrared technology. Measurements of the developed gas analyzer were in good agreement with those of the commercial instrument, and its accuracy was comparable. Therefore, the TDLAS sensor has strong application prospects in atmospheric CO_2_ and H_2_O concentration detection and ecological soil flux monitoring.

## 1. Introduction

There is an increasing focus on global warming and environmental problems as a result of rising greenhouse gas (GHG) emissions. CO_2_ and H_2_O are considered two important GHGs. CO_2_ concentration has increased from 270 ppm in 1750 [1] to 470.5 ppm in 2019 [2]. China’s CO_2_ emissions reached 11,255.88 million tons in 2018, accounting for approximately 30% of global emissions [3]. However, since the COVID-19 outbreak in 2019, CO_2_ emissions have slightly decreased because of fewer human activities in China [4]. Monitoring the change in CO_2_ concentration can provide data support for formulating carbon emission reduction policies. Thus, developing a portable CO_2_ and H_2_O sensor with good stability, high precision, and a short response time is highly significant and has practical value.

Optics-based methods have several advantages and are widely used in trace gas monitoring. Non-dispersive infrared (NDIR) has been increasingly applied to atmospheric CO_2_ and H_2_O monitoring [5,6,7]. However, the application of NDIR is limited because it uses a broadband light source and therefore has poor selectivity. Moreover, it is easily influenced by the presence of other gases. Cavity-ring down spectroscopy (CRDS) and off-axis integrated cavity output spectroscopy (OA-ICOS) have also been increasingly applied for the continuous monitoring of atmospheric CO_2_, CH_4_, and H_2_O [8,9,10,11,12,13]. Although CRDS and OA-ICOS sensors have high sensitivity, they require relatively complex setups with laser frequency locking to longitudinal cavity resonances [14]. Tunable diode laser absorption spectroscopy (TDLAS) also has advantages, such as high sensitivity, high selectivity, and fast response [15,16,17]. It is widely used and has many applications, such as industrial process control, atmospheric GHGS monitoring, breath diagnostics, combustion diagnosis, deep-sea dissolved gas detection, and isotope detection [18,19,20,21]. CO_2_ and H_2_O concentrations in air are several hundred and tens of thousands of parts per million (ppm), respectively; thus, the detection sensitivity of TDLAS is sufficient for CO_2_ and H_2_O monitoring. Moreover, TDLAS systems are less complex and expensive than CRDS- and OA-ICOS-based systems [22]. Typically, two different methods are used to conduct TDLAS, namely direct absorption spectroscopy (DAS) and wavelength modulation spectroscopy (WMS). In terms of noise, it is always randomly distributed in all frequency bands in DAS [23], while in WMS, there is better noise suppression and sensitivity.

Most contemporary TDLAS systems are based on LabVIEW (National Instruments, USA); these use a commercial laser driver to control laser temperature, a function signal generator to generate a driving signal, a lock-in amplifier to generate a demodulation signal, a date acquisition card to collect the detector signal, and a laptop to process the signals [19,24,25,26]. As a result, the gas sensing system is very large and consumes a great deal of power. Luo et al. used a laser controller (Arroyo 6305), data acquisition card (NI 9223), and function generator (RIGOL 4102) to measure gas temperature in a LabVIEW-based TDLAS system [27]. Zhang et al. used a commercial laser controller (CLD1015, Thorlabs), lock-in amplifier (LIA-MV-200-H, FEMTO Messtechnik GmbH, Berlin, Germany), and industrial computer (PCM-3365, Advantech Technology, China) to measure CO_2_ using a TDLAS system [14]. Chang et al. developed an oxygen detection system using a laptop equipped with a DAQ card (USB-6211;NI) and a LabVIEW software platform [28]. In addition, the study of miniaturized multi-pass cells (MPCs) can also improve the integration of the system [29,30]. There were some self-developed laser drivers and LIAs in industrial TDLAS sensing [31,32]; however, the laser driver and LIA were two separate modules that occupied a larger space.

To solve this problem, we designed a TDLAS-WMS-based portable gas sensing system for monitoring CO_2_ and H_2_O concentrations. Laser drive signal generation, detector signal acquisition, and computer signal processing functions (signal demodulation), all integrated into a small circuit, were realized using an STM32 microcontroller. Furthermore, we designed a small MPC to achieve a miniaturized and lightweight system with low power consumption. The external size of the MPC chamber was 34 mm × 34 mm × 125 mm, and it was made of stainless steel. The volume and weight of the MPC chamber were only 52 mL and 0.75 kg, respectively. Using this MPC, CO_2_ and H_2_O optical paths of 430 cm and 10.7 cm, respectively, were obtained. The two optical paths were integrated into a single MPC. The total weight and power consumption of the system were only 5 kg and 10 W, respectively. The sensors used 2004 nm and 1392 nm DFB lasers to monitor the atmospheric concentrations of CO_2_ and H_2_O. We also compared our measurements with those of commercial NDIR instruments to verify long-term concentrations.

## 2. Materials and Methods

### 2.1. TDLAS-WMS Theoretical Principle

In WMS, the frequency of light, v(t), is determined using a slow scan, $v_{scan}\left(t\right)$, with fast sinusoidal modulation at amplitude, A, and frequency, $f_{m}$ (Equation (1)):

(1) $$\begin{array}{c} v\left(t\right)=v_{scan}\left(t\right)+A\cos\left(2\pi f_{m}t\right) \end{array}$$

According to the Beer–Lambert law, the laser intensity transmitted through an absorbing medium of temperature, T, and pressure, P, can be expressed using Equation (2):

(2) $$\begin{array}{c} I\left(t\right)=I_{0}\left(t\right)\exp\left[-\alpha \left(v\right)\right]=I_{0}\left(t\right)\exp\left[-S\left(T\right)\phi_{v}PX_{abs}L\right] \end{array}$$

where $I_{0}\left(t\right)$ is the background laser intensity without absorption, and α(v) represents the absorption, which depends on the mole fraction of the absorbing species, $X_{abs}$, absorption path length, L, and transition line strength, S(T), with associated line–shape function, $\phi_{v}$. A lock-in amplifier extracted nf-WMS signals using Equation (3):

(3) $$\begin{array}{c} WMS_{nf}=I\left(t\right)\cos\left(2\pi nf_{m}+\theta \right)⊗LPF \end{array}$$

where *LPF* indicates a low-pass filter, and θ denotes the phase shift. A Butterworth low-pass filter was applied in the current system. Using a digital lock-in, any *nf*-WMS component within the bandwidth of the data acquisition system could be demodulated. Laser output power and the intensity of light received by the detector were influenced by the environment, which led to deviations in the calculated results during the field test; thus, it is crucial to normalize light intensity. Variations in laser power and instrumentation effects were eliminated by normalizing the 2*f*-WMS signal with the amplitude of a modulated sinusoidal signal without absorption, $DS_{sine}$ [33]. The measured concentration value, $X_{abs}$, is expressed using Equation (4):

(4) $$\begin{array}{c} \begin{array}{c} X_{abs}\propto WMS_{2f}/DS_{Sine} \end{array} \end{array}$$

### 2.2. Selection of CO_2_ and H_2_O Absorption Lines

**Figure 1:**
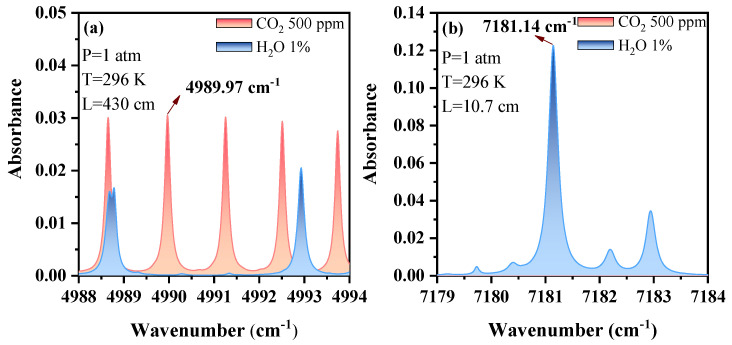
Simulated absorbance of CO_2_ and H_2_O in the selected wavenumber range (**a**) 4988–4994 cm^−1^ and (**b**) 7179–7184 cm^−1^ with the HITRAN database.

CO_2_ and H_2_O have several strong absorption bands from 1 μm to 2.5 μm. We performed a spectral simulation to determine whether the selected lines had sufficient strengths for measurement. The CO_2_ absorption bands near 1.57 μm and 2.0 μm were free of H_2_O interference and could be used for CO_2_ measurements. The line strength of CO_2_ near 2.0 μm was much stronger than that near 1.57 μm. Figure 1a,b show the simulation of spectra absorption at approximately 4991.25 cm^−1^ and 7181.14 cm^−1^ based on the HITRAN 2016 database for 500 ppm CO_2_ and 1% H_2_O, respectively, under nominal conditions (pressure = 1 atm and temperature = 296 K). The measurements of the two gases did not show any mutual effects. Thus, the target lines were suitable for the detection of CO_2_ and H_2_O without any serious interference. The absorption lines of CO_2_ and H_2_O (centered at 4989.97 cm^−1^ and 7181.14 cm^−1^, respectively) were selected for further research. DFB lasers with wavelengths of 2004 nm (DFB-2004, nanoplus GmbH, Gerbrunn, Germany) and 1392 nm (NLK1E5EAAA, NEL, Oslo, Norway) were used.

### 2.3. Experimental Setup

**Figure 2:**
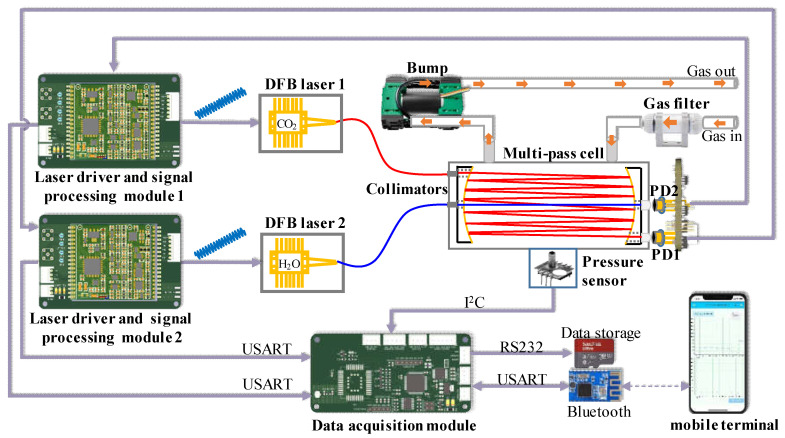
Schematic diagram of the TDLAS system for measuring atmospheric CO_2_ and H_2_O concentrations.

The developed TDLAS system, depicted in Figure 2, comprised three functional parts, namely an optical part, an electrical part, and a gas circuit. All parts were fitted in a portable carrying case (height = 134 mm, length = 388, and width = 290 mm). The optical part comprised a small self-designed MPC, two optical fiber collimators, and two plano-convex lenses. The electrical part comprised two laser drive circuits, a data acquisition circuit, and a lithium battery pack (18650, Panasonic). The gas circuit part comprised a gas inlet, a gas inlet filter (10 μm), a vacuum pump, and an outlet pipe.

The laser drive circuit was equipped with a current drive and laser temperature control. First, the digital signals of sawtooth (40 Hz) and high-frequency sine (5 kHz) waves generated by the microprogrammed control unit (MCU) were converted into analog voltage signals using a digital-to-analog converter (DAC). Then, a current signal with a center current of 70 mA and scanning range of ±50 mA was generated using a voltage-to-current circuit to realize current drive in the laser. The MCU simultaneously communicated with the DAC (MCP4726) through the I^2^C interface to ensure that the DAC output a constant voltage, which was the set temperature parameter. Thereafter, the temperature control chip (Maxim, MAX8521) adjusted the output current to the thermoelectric cooler (TEC) through proportional integral and differential control to realize temperature control of the laser. The two lasers generated optical signals with central wavelengths of 1392 nm and 2004 nm under the combined action of the laser current drive and temperature control. The modulated optical signal was reflected several times in the self-designed MPC. The light signal from the MPC was passed through the convex lens and focused on the photodetector (PD), thereby converting it into a current signal. The current signal was then converted into a voltage signal using a trans-resistance amplifier. The voltage signal was converted using an analog-to-digital converter (ADC) and collected by the MCU, following which a digital lock-in amplifier (DLIA) was used in real time in the MCU. The second harmonic signal was obtained using a BLPF. The normalized second harmonic signal was positively correlated with the target gas concentration, thus realizing the demodulation of the signal. The MCU transmitted the demodulated data to the data acquisition module through a serial port. The data acquisition module transmitted the concentrations of CO_2_ and H_2_O as well as the data regarding light intensity, temperature, and pressure to a Bluetooth module and stored these in a secure digital (SD) memory card. These data can be observed in real time through a mobile application software or read on the SD card.

**Figure 3:**
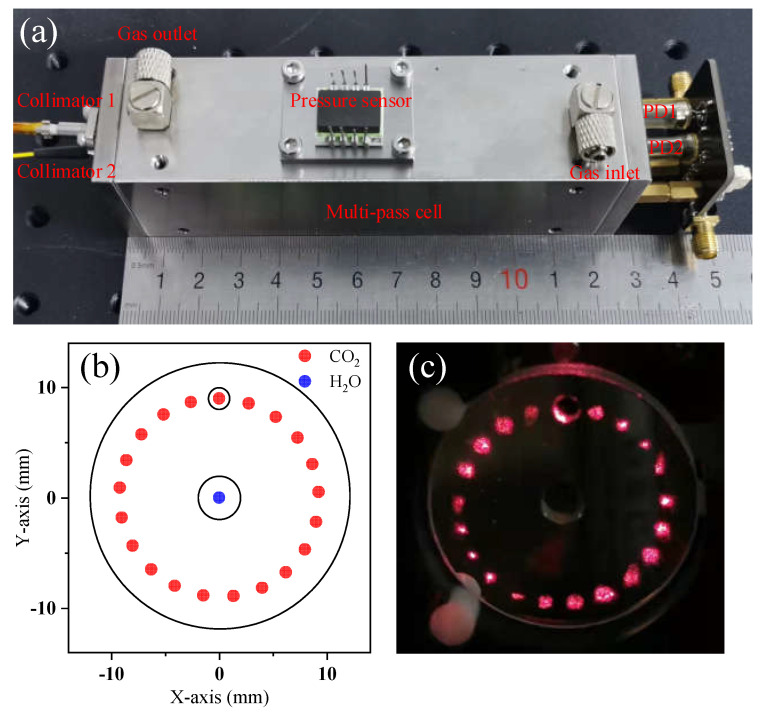
Homemade double light path miniaturized Herriott MPC, (**a**) photograph of the MPC structure, and (**b**,**c**) spot distribution on the simulation and mirrors, respectively.

Figure 3 shows the homemade dual optical path miniaturized MPC, which comprised a Herriott cell structure with two parallel concave mirrors [34]. It was made of solid stainless steel. Countersunk holes were machined on both sides of the lens. The lenses then were pressed onto an O-shaped rubber ring and screwed into place with two stainless steel squares at each end. The compact optical system formed a circular distribution of light spots on the mirror. Optical transmission matrix theory was used to simulate optical path propagation in the MPC. For the lens, diameter, thickness, and curvature radius were 25 mm, 3 mm, and 614.9 mm, respectively. There was a CO_2_ light-passing hole with a diameter of 2.3 mm at the edge of the lens and a H_2_O light-passing hole with a diameter of 4 mm at the center of the lens. By adjusting the distance between the two lenses and the angle of the incident light, a different light spot distribution was obtained on the mirror, thus achieving different light paths. Considering the required effective optical path and portability, we set the distance between the two lenses to 10.7 cm. The spot distribution of the simulated CO_2_ and H_2_O optical paths is shown in Figure 3b. The CO_2_ optical path reflected 40 times, with an effective optical path of 430 cm, and the H_2_O optical path had a single optical path, with an effective optical path of 10.7 cm. Figure 3c shows the spot distribution on the actual mirror; the theoretical spot distribution was in good agreement with the actual spot distribution. In the experiment, two optical paths were integrated into a single MPC. Two optical fiber collimators, a pressure sensor, and two photodetectors were integrated outside of the absorber chamber. The laser beam was coupled with the MPC by a fiber collimator. The light beam was reflected several times in the MPC and then received by two photodetectors at the MPC outlet. A picture of the MPC is shown in Figure 3a.

**Figure 4:**
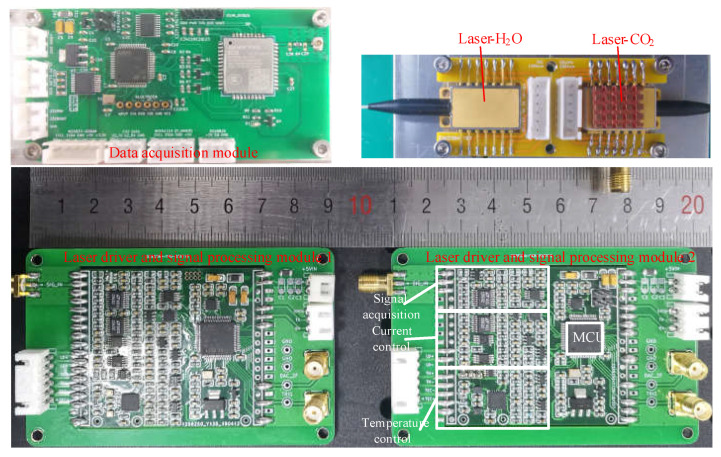
Actual circuit board of the miniaturized TDLAS system.

Figure 4 shows a picture of the actual circuit boards of the miniaturized TDLAS system. The laser drive and signal process module (size 90 mm × 45 mm) realized the functions of current control, laser temperature control, signal acquisition, and demodulation, with a temperature control accuracy of ±1.4 mK and a current control accuracy of ±0.5 μA. The MCU drove the superposition signals of 40 Hz low-frequency sawtooth waves and 5 kHz sine waves generated by the DAC; then, it realized current control of the laser through the volt-to-current circuit. Furthermore, the module collected the voltage signal converted by the photodetector through the ADC, and it carried out DLIA in real time, along with the fourth-order Butterworth low-pass filter function. These calculations were realized in the MCU, which also improved system integration and ensured a more portable system. The data acquisition module collected the real-time gas concentration data and ambient temperature and pressure parameters obtained from the demodulation of the two laser control panels; then, these data were stored on the SD card and transmitted to a computer through RS232 or to a mobile phone terminal through the Bluetooth module for online monitoring.

## 3. Results and Discussion

### 3.1. Calibration and Measurement Precision

**Figure 5:**
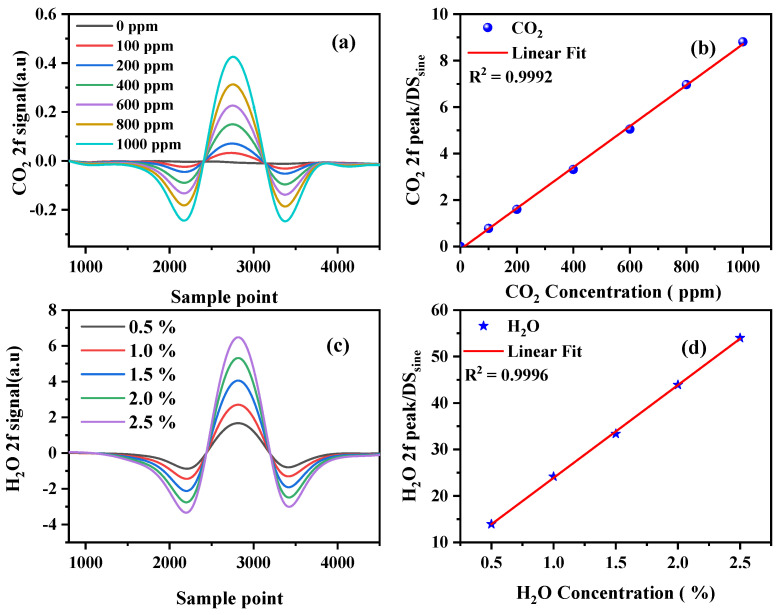
Second harmonic signal at different concentrations of (**a**) CO_2_ and (**c**) H_2_O. Calibration measurements of the TDLAS system for (**b**) CO_2_ and (**d**) H_2_O.

To evaluate the linear response characteristics of the developed TDLAS sensor, we used a commercial gas distribution system (Environics 4000), a standard concentration of 1000 ppm CO_2_ gas cylinder, and a high-purity (99.99%) nitrogen cylinder to provide the required gas samples. During the test, high-purity nitrogen and 1000 ppm CO_2_ were configured with different CO_2_ concentrations (0, 100, 200, 400, 600, 800, and 1000 ppm) through the gas distribution system. We used a dew-point generator mixed with high-purity nitrogen to produce different H_2_O concentrations (0.5%, 1%, 1.5%, 2%, and 2.5%). The configured gas was passed into the gas chamber of the sensor at a flow rate of 500 mL/min. Figure 5a,c show the measured normalized 2f signals for different concentrations. As shown in Figure 5b,d, the system response values showed a linear correlation with the standard gas value of the input; the linear regression R^2^ was >0.999, indicating that the system had a good linear response.

**Figure 6:**
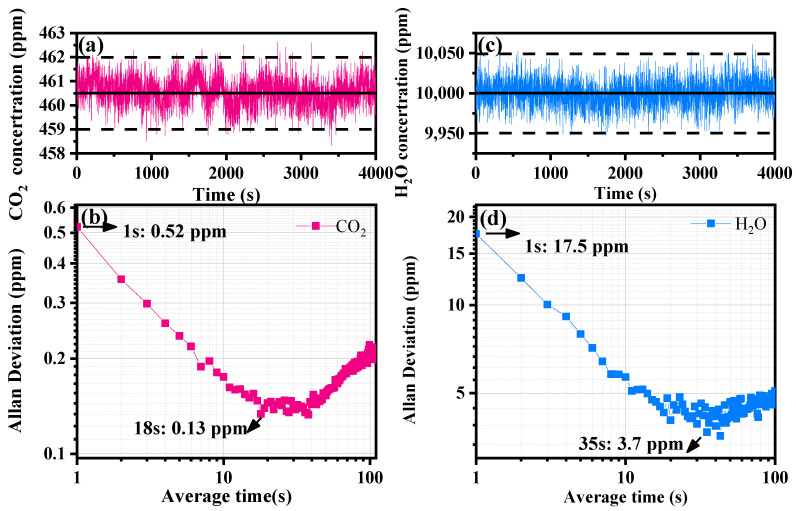
(**a**,**c**) Standard CO_2_ and H_2_O concentrations at 460.5 ppm and 10,000 ppm, respectively, tested for 4000 s; (**b**,**d**) Allan deviation analyses of CO2 and H_2_O, respectively, with detection limits as a function of averaging time.

The accuracy and stability of a system were measured by testing the data results of known CO_2_ and H_2_O concentrations over a specific period, and these results were analyzed using the Allan variance method. As shown in Figure 6, we tested 460.5 ppm CO_2_ and 10,000 ppm H_2_O for 4000 s. The Allan variance is plotted on a log–log scale in Figure 6b,d. The measurement accuracies were 0.52 ppm and 17.5 ppm at 1 s integration time for CO_2_ and H_2_O, respectively; the limits of detection for CO_2_ and H_2_O were 0.13 ppm and 3.7 ppm at integration times of 18 s and 35 s, respectively. Figure 6b,d also show that when white noise was the main noise, with increases in integration time, the Allan variance value decreased. The Allan variance first decreased mainly because white noise was the dominant noise of the system and then fell to the lowest point, which meant that it reached the detection limit of the system. Then, as the 1/f noise was dominant, it rose. When the system dominant noise was 1/f noise, increasing the integration time did not improve the accuracy of the system. For real-time measurements of CO_2_ and H_2_O concentrations, when the accuracy at an average time of 1 s did not meet the requirements, the measurement accuracy was improved by increasing the integration time. It should be noted that the balance between measurement accuracy and system time resolution should also be considered.

### 3.2. System Response Time

**Figure 7:**
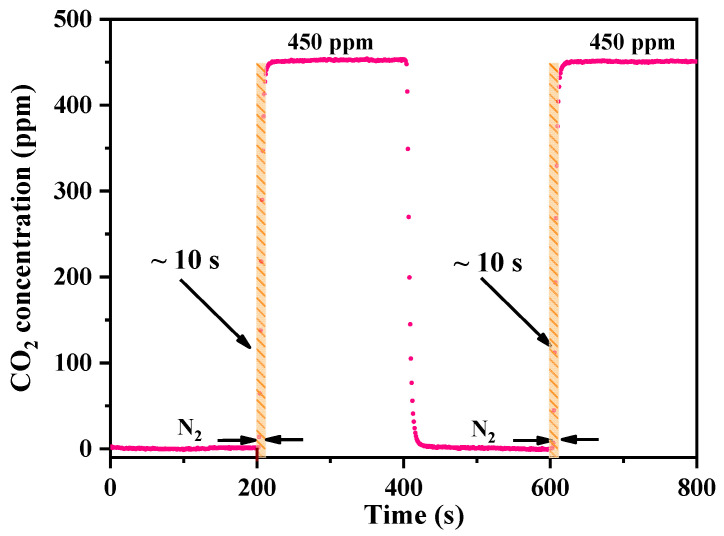
Response time of the miniaturized TDLAS system.

For online and real-time measurements of concentration, the flow response time of a sensor is a crucial parameter because faster instrument response implies a high temporal resolution, low data latency, and highly accurate real-time monitoring. This is attributed to the MPC structure, length of the gas circuit, and gas flow rate. The volume of the designed MPC was 52 mL, which is smaller than that of conventional MPCs. To examine the signal response process, we used a mass flowmeter at a fixed flow rate of 500 mL/min. First, the closed chamber was flushed with high-purity nitrogen and then filled with a 450 ppm CO_2_/N_2_ mixture until a steady-state concentration was reached. Finally, high-purity nitrogen was injected again into the closed cavity, and this test was repeated multiple times. As shown in Figure 7, we analyzed the system response time (0–90% rise time) twice, and it was approximately 10 s.

### 3.3. Comparison and Field Measurements

**Figure 8:**
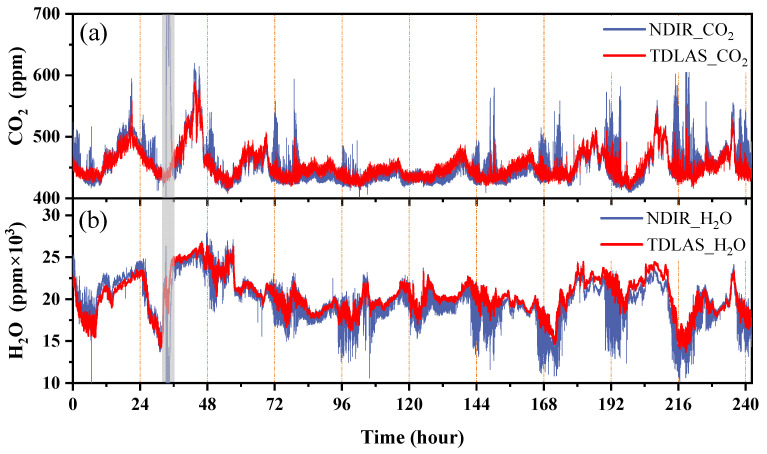
Comparison of (**a**,**b**) CO_2_ and H_2_O concentrations, respectively, for approximately 10 days.

The sensor was evaluated by comparison monitoring on a balcony outside of the laboratory using a commercial NDIR-based instrument (LI-7500). With continuous day and night monitoring, the experiment was conducted from 17 to 27 August 2022 (~240 h sampling) at the Science Island, Hefei City, China. We ensured the consistency of the gas samples by placing the TDLAS inlet pipe near the LI-7500 probe head. Figure 8 shows the results of the long-term comparison between the developed TDLAS device and the commercial NDIR-based device. The red and blue parts show the data of the two sensors, respectively. The open-path NDIR device (LI-7500) showed merits in terms of lower data delay and a high ability to capture concentration variations in high-speed airflow compared with our close-path system; therefore, there were more peak characteristics in its measurement. The close-path TDLAS system had a gas path delay, which had a low-pass filtering effect on gas concentration measurement. Figure 8a,b show the comparison data of CO_2_ and H_2_O concentrations, respectively, for 240 h. As shown in Figure 8a, CO_2_ concentration in the air increased at night and decreased during the day every day. This was because CO_2_ concentration in the air increased due to plant respiration at night and decreased as plants resumed photosynthesis during the day. As shown in Figure 8b, water vapor in the air showed no significant changes, and it fluctuated within a range of 1.5–2.5%. As shown in Figure 8, there was good consistency between the results of the TDLAS and NDIR devices.

## 4. Conclusions

In this study, we developed a portable TDLAS-based gas analyzer that can simultaneously detect CO_2_ and H_2_O concentrations in the air. The total weight of the developed instrument was 5 kg, and its volume was 388 mm × 290 mm × 134 mm. First, we designed a small MPC (volume = 34 mm × 34 mm × 125 mm), which ensured better system integration. A TDLAS drive module was designed to achieve a high-precision current and temperature control of DFB lasers with a temperature control accuracy of ± 1.4 mK and a current control accuracy of ± 0.5 μA. The module realized the function of DLIA and signal processing and thus realized the demodulation of the harmonic signal. The size of a single module was only 90 mm × 45 mm. It also improved the integration of the system. Then, we used 2004 nm and 1392 nm DFB lasers to measure CO_2_ and H_2_O concentrations in the air, respectively; the two optical paths were integrated into a single MPC. The effective optical paths of CO_2_ and H_2_O were 430 cm and 10.7 cm, respectively, and the system detection limits were 0.13 ppm and 3.7 ppm at integration times of 18 s and 35 s, respectively. The developed TDLAS system was compared with an NDIR-based commercial instrument for 240 h, and the results showed good agreement between the results of the two instruments. Thus, the developed equipment has broad application prospects for measuring CO_2_ and H_2_O concentrations in the air and for monitoring ecological soil flux.

## Data Availability

Data sharing not applicable.
